# Integrating combining ability and multivariate analysis for the selection of superior tomato hybrids

**DOI:** 10.3389/fpls.2026.1788062

**Published:** 2026-04-10

**Authors:** Tâmara K. C. Mendes, Mauricio S. Araújo, Wanderson M. B. Silva, Francisca A. S. Oliveira, Denizard A. S. Bueno, Ademir M. Lima, João V. M. Nunes, Jéssica E. F. Santos, Leonardo L. Bhering, Derly J. H. Silva

**Affiliations:** 1 Department of Agronomy, Federal University of Viçosa, Viçosa, Minas Gerais, Brazil; 2 Department of Genetics, Luiz de Queiroz College of Agriculture, University of São Paulo, Piracicaba, São Paulo, Brazil; 3 Department of Biology, Federal University of Viçosa, Viçosa, Minas Gerais, Brazil

**Keywords:** fruit quality, general combining ability, multi-trait selection, North Carolina II, *Solanum lycopersicum* L.

## Abstract

**Introduction:**

Tomato is a globally important crop and the second most consumed vegetable worldwide, with an estimated global production of 188 million tons annually. However, high-yield production systems increase vulnerability to pests and diseases, posing major challenges for tomato breeding programs. This study aimed to evaluate combining ability and identify superior Italian-type tomato hybrids by integrating a North Carolina II (NCII) mating design with multi-trait selection using the multi-trait genotype–ideotype distance index (MGIDI).

**Methods:**

Nine inbred lines previously characterized for resistance alleles were crossed in a 4 × 5 factorial scheme, generating 20 hybrids, which were evaluated together with their parents and a commercial check in a randomized complete block design with three replications. Traits related to plant architecture, yield, and fruit quality were assessed.

**Results and discussion:**

Combining ability analysis based on the NCII design revealed significant effects of specific combining ability (SCA) (*p* < 0.01) for most plant architecture traits and for productivity per plant (PP), with SCA mean squares exceeding those of general combining ability (GCA) for key traits, indicating the predominance of non-additive genetic effects. By contrast, internode diameter (ID) and fruit size (FS) showed stronger additive contributions, with broad-sense heritability estimates of 60.42% and 82.95%, respectively. In the hybrid evaluation, all plant architecture traits showed significant genotypic effects (*p* < 0.01), with heritability ranging from 64.29% to 85.85%. Yield-related traits were also highly significant (*p* < 0.01), with heritability values ranging from 67.75% to 87.03%, and several hybrid combinations outperformed the commercial check for yield per plant. The MGIDI analysis retained three factors (eigenvalues > 1), explaining a substantial proportion of the total variation, and identified hybrids H17, H2, and H11 as the closest to the ideotype, combining favorable plant architecture, fruit quality, and yield. Overall, these results highlight the breeding potential of the evaluated inbred lines and indicate the need to validate the selected hybrids in multienvironment trials prior to recommendation.

## 1 Introduction

Tomato (*Solanum lycopersicum* L.) is one of the most widely cultivated vegetable crops worldwide and ranks as the second most consumed vegetable (Zhu and Miller, 2025), with an estimated global production of 188 million tons annually (FAOSTAT, 2025). Its importance is associated with high market demand and wide adaptability across production systems, in addition to its nutritional and functional value, as tomato fruits are rich in dietary fiber, vitamin C, sugars, phenolic compounds, and lycopene (Choi et al., 2014; Salehi et al., 2019). From an agronomic perspective, tomato yield and adaptation are strongly influenced by environmental conditions, particularly high temperatures that frequently occur during the cropping season (Kumar et al., 2020). Heat stress during critical growth stages, such as flowering and fruit set, can reduce fruit number, fruit weight, and overall yield, compromising production efficiency and economic returns. These limitations are especially relevant in tropical and subtropical regions, where temperature increases intensify genotype performance variability, reinforcing the need for agronomic and breeding strategies focused on selecting genotypes with superior performance and adaptability under high-temperature conditions (Visakh et al., 2026).

High yield demand, however, makes tomato cultivation particularly susceptible to a wide range of biotic stresses, including pests and diseases (Kawicha et al., 2023). As a result, the development of improved cultivars combining resistance to major pathogens and desirable agronomic traits has become a priority in breeding programs (Manjunath et al., 2025). Traits such as fruit firmness, color, shape, pH, soluble solids content (^°^Brix), and titratable acidity are of particular importance for both fresh market and processing sectors. These traits can be improved through the selection of superior parental genotypes, allowing genetic gains in fruit quality and performance (Matos et al., 2021; Brake et al., 2022). Soluble solids content is a key indicator of sweetness and flavor, while titratable acidity, commonly expressed as citric acid percentage, contributes to the organoleptic balance. Both are largely influenced by additive and non-additive genetic action, as well as by environmental influence. Thus, these traits can be efficiently improved through targeted selection (Glogovac et al., 2022b).

The incidence of diseases caused by fungi, bacteria, viruses, and nematodes remains a major constraint to sustainable tomato production. In open-field systems, disease epidemics are frequently driven by leaf wetness and high humidity, which favor infection and rapid pathogen spread, particularly for foliar diseases. In addition, several pathogens can persist between seasons in crop residues and act as primary inoculum, with dispersal enhanced by rain splash and wind, making disease pressure recurrent in successive cycles. Disease management in intensive production systems remains challenging due to the limited availability of effective control methods. Therefore, integrated strategies that reduce canopy moisture, including adequate plant spacing, staking and pruning, weed control, drip irrigation, mulching, and, when feasible, protected cultivation (e.g., tunnels/high tunnels), can substantially delay disease onset and mitigate yield losses. In this context, breeding for disease resistance is essential (Padilla-Hurtado et al., 2022; Zhao et al., 2022). Incorporating resistance genes into elite backgrounds while preserving key agronomic attributes (fruit quality, plant architecture, and yield potential) increases cultivar adaptability under high disease pressure and reduces dependency on external inputs. This strategy also contributes to improved yield stability and economic returns (Egea et al., 2022).

Hybrid breeding is the most widely adopted strategy in tomato breeding, as it enables the effective exploitation of genetic variability and heterosis while facilitating the accumulation of favorable alleles associated with resistance, vigor, and agronomic performance (Shafqat et al., 2021). Through artificial pollination, directed crosses between carefully selected parental lines can be performed, allowing breeders to generate hybrids with superior yield and adaptation across diverse production environments (Lopez-Gomollon et al., 2022; Zeist et al., 2022). In this context, parental choice represents a central determinant of hybrid success, since the genetic contribution of each parent directly influences hybrid expression. The factorial crossbreeding scheme based on the North Carolina II design provides a robust and systematic framework for evaluating parental performance, dividing genetic effects into general combining ability (GCA) and specific combining ability (SCA), which together capture the additive and non-additive components underlying hybrid performance (Coelho et al., 2020; Chikh-Rouhou et al., 2024; Ferreira et al., 2024). GCA reflects the average performance of a genotype across hybrid combinations and is particularly relevant for identifying parents with consistent and favorable genetic contributions, whereas SCA highlights specific parental combinations that express superior performance due to dominance and epistatic interactions, offering valuable insights for the development of high-performing tomato hybrids (Falconer and Mackay, 1996).

Although GCA analysis provides useful information about the potential for parental matching, its effectiveness is reduced when mating decisions require the simultaneous consideration of multiple correlated traits (Lynch et al., 1998). To address this challenge, multi-trait selection strategies have been developed (Araújo et al., 2024; Graci et al., 2025; Retore et al., 2026), including multi-trait selection indices such as the multi-trait genotype–ideotype distance index (MGIDI) (Olivoto et al., 2022), which enables the identification of genotypes that most closely approach a predefined ideotype aligned with specific breeding objectives. Therefore, the present study aimed to quantify both additive and non-additive genetic effects in Italian-type tomato inbred lines using a North Carolina II (NCII) mating design, allowing the estimation of general and specific combining abilities and their respective variance components. Furthermore, the MGIDI index was applied to jointly integrate traits related to plant architecture, fruit quality, and yield, supporting the identification of superior tomato hybrids with balanced agronomic performance and desirable traits for the fresh fruit market.

## 2 Materials and methods

### 2.1 Genetic material

A total of 200 tomato inbred lines were obtained from the Vegetable Germplasm Bank of the Federal University of Viçosa (UFV). These lines were developed through single-seed descent (SSD) over eight generations. The inbred lines originated from a cross between the commercial hybrids Mascot F1 (cherry type) and Santyno (salad type), both from Agristar. All inbred parental lines were genotyped using single nucleotide polymorphism (SNP) markers linked to disease resistance genes by the Vegetable Breeding Program at UFV. The markers were associated with resistance to *Meloidogyne incognita* (*Mi-1*), *Fusarium oxysporum* f. sp. *lycopersici* races 1, 2, and 3 (*I-2, I-3, I-7*), *Verticillium* spp. (*Ve*), tomato spotted wilt virus (*Sw-5, Sw-7*) (Oliveira et al., 2018; Lahre et al., 2023), *Fusarium crown* and root rot (*Frl*) (Fazio et al., 1999), tomato yellow leaf curl virus (*Ty-1, Ty-2*), and tomato mosaic virus (*Tm1, Tm2, Tm2a*) (Panthee et al., 2013). These molecular characterization data were generated by the Vegetable Breeding Program at UFV as part of ongoing projects and have not been previously published. Based on the allelic profiles, nine Italian-type inbred lines were selected for hybridization. The resistance classification (Susceptible (S), Resistant (R), Heterogeneous (H), and Unknown (U)) derived from these markers is provided in **Supplementary Table 1** and is presented solely to document the criteria adopted for parental line selection in the present study.

The crosses were structured according to the North Carolina II (NCII) mating design, as described by Comstock and Robinson (1952). Two groups of parents were defined: a male group composed of four inbred lines and a female group composed of five inbred lines. Each male parent was crossed with all female inbred lines, generating a total of 20 hybrids derived from a factorial combination (4 × 5). The donor parents carried a higher number of resistance alleles, whereas the recipient parents were more susceptible. The choice of the NCII method was based on the prior separation of the parental lines into two contrasting genetic groups according to their resistance allele profiles. This strategy enabled a factorial crossing scheme that efficiently estimates additive and dominance variance components. Compared with a full diallel scheme, this approach considerably reduces the number of crosses required while maintaining robust inference regarding combining ability, which is highly relevant in hybrid breeding programs. The field experiment was conducted at the Teaching, Research, and Extension Unit (UEPE – Horta Velha) (20^°^45^′^14^′′^ S, 42^°^52^′^53^′′^ W; 648.74 m above sea level), of the Universidade Federal de Viçosa (UFV), located in Viçosa, Minas Gerais, Brazil. Laboratory analyses were performed at the Laboratory of Genetic Resources Management and Vegetable Crop Breeding, Department of Agronomy, UFV.

### 2.2 Hybrid seed production

Hybridization was carried out in a greenhouse starting from inflorescence emergence. Recipient flowers were manually emasculated two days before anthesis to prevent self-pollination and subsequently received pollen from donor plants. After fruit maturation, the fruits were harvested and subjected to maceration followed by controlled fermentation for 48 hours to remove the mucilage adhering to the seeds. The seeds were then washed and dried using a centrifuge developed by UEPE – Horta Velha staff members. After 48 hours of drying, the seeds were stored under controlled conditions.

### 2.3 Field evaluation of plant architecture traits and yield

A total of 30 genotypes were evaluated, including 20 hybrids derived from the crosses, nine parental lines, and one commercial reference cultivar (*Monza* F1, saladette type). The experiment was conducted using a randomized complete block design (RCBD) with three replications. Each block consisted of three rows, each 17 m in length, with 0.6 m spacing between plants and 1.0 m between rows. Each experimental plot comprised six plants per genotype, with the three central plants used for phenotypic evaluations.

The evaluated traits were grouped into three categories: plant architecture, yield components, and fruit quality. Traits related to plant architecture and yield included the number of fruits per plant (NFP), calculated as the total count of fruits up to the fifth truss, considering both the main stem and secondary stems; yield per plant (YPP, kgplant^−1^), obtained by dividing the total plot yield by the number of evaluated plants (three); yield per area (YPA, kgm^−2^), calculated by dividing YPP by the plot area (0.6m^2^); and estimated yield (EYLD, tha^−1^), derived by multiplying YPA by 10. Leaf length and width were measured using a measuring tape, and internode diameter and length were assessed using a flexible ruler.

### 2.4 Laboratory analysis of fruit quality traits

Fruit quality traits were measured at physiological maturity using eight fruits collected from the third and fourth trusses of the three central plants in each plot. Fruit size (FS) was determined as the average of the equatorial and polar diameters measured in four fruits per genotype. Pericarp firmness (PF) was assessed using a penetrometer, with bilateral readings taken from four fruits. Acidity (pH) was evaluated using a benchtop pH meter on a homogenized sample composed of eight fruits. Total soluble solids content (TSS, °Brix) was measured using a digital refractometer. Titratable acidity (TA) was determined by titrating 5 g of pulp diluted in 100 mL of deionized water with 0.005 mol L^−1^ NaOH until the appearance of a persistent pink coloration. The TSS/TA ratio was used as an indicator of fruit flavor and ripening stage (Rasul et al., 2022; Tahir et al., 2022, 2023). Each measurement was performed twice per plot, and mean values were calculated using *R* (R Core Team, 2025).

### 2.5 Statistical analysis

The statistical models used in this study are described in Equations 1–10. Equation 1 presents the linear model of the NCII design, while Equations 2–4 describe the procedures for estimating the variance components associated with genetic effects and parent interaction. Equations 5–8 refer to the calculation of derived genetic parameters, including additive and non-additive variances, as well as heritability estimates. Finally, Equations 9, 10 present the expressions used to obtain complementary parameters and auxiliary metrics employed in the interpretation of results and in the selection of superior genotypes.

#### 2.5.1 Analysis of GCA and SCA based on the NCII design

The evaluation of the potential for combining parental genotypes for plant breeding purposes was carried out following the North Carolina II design (Comstock and Robinson, 1952). The North Carolina II factorial scheme was adopted because it allows simultaneous estimation of general combining ability (GCA) and specific combining ability (SCA) effects, with clear partitioning of additive and non-additive genetic variances, while maintaining a balanced and operationally feasible crossing structure for the number of parental lines involved (Hallauer et al., 2010). In this stage, a two-way ANOVA was considered, and the effects of males and females and of interaction were considered random so that the variance components would have legitimacy. In this design, the parents are organized into two groups (male and female), and each male parent is mated with all female parents. This approach allowed for the estimation of the effects of GCA on the parental lines and the effects of SCA on the hybrids, as described in the Equation 1:

(1) $$Y_{ijk}=\mu +M_{i}+F_{j}+MF_{ij}+B_{k}+E_{ijk}$$

where *Y_ijk_* represents the observed phenotypic value of the *k*-th progeny from the cross between the *i*-th male and the *j*-th female; *µ* is the overall mean; *M_i_* is the random effect of the *i*-th male parent; *F_j_* is the random effect of the *j*-th female parent; *MF_ij_* is the specific interaction effect between the *i*-th male and the *j*-th female, representing the specific combining ability (SCA); *B_k_* is the block effect; and *E_ijk_* is the residual error term.

Based on the variance components obtained from Model (1), the narrow-sense heritability (*h*^2^) was estimated considering that, under the North Carolina II design and assuming absence of epistasis and maternal effects, the variance among male (or female) parents corresponds to one-quarter of the additive genetic variance. Thus, the additive genetic variance was estimated as $\sigma_{A}^{2}=4\sigma_{M}^{2}$ (or $4\sigma_{F}^{2}$), and the dominance variance as $\sigma_{D}^{2}=4\sigma_{MF}^{2}$. Narrow-sense heritability was then calculated as:

(2) $$h^{2}=\frac{\sigma_{A}^{2}}{\sigma_{A}^{2}+\sigma_{D}^{2}+\sigma_{E}^{2}}$$

where $\sigma_{A}^{2}$ is the additive genetic variance, $\sigma_{D}^{2}$ is the dominance variance, and $\sigma_{E}^{2}$ is the residual variance, all derived from the expected mean squares of Model (1).

#### 2.5.2 Hybrid evaluation

Heterosis (*h*) was calculated as the percentage deviation of the hybrid mean (*F*_1_) from the mid-parent value, defined as the average performance of the two parents (*P*_1_ and *P*_2_):

(3) $$h(\%)=(\frac{F_{1}-\frac{P_{1}+P_{2}}{2}}{\frac{P_{1}+P_{2}}{2}})\times 100$$

Heterobeltiosis (*H_b_*) was estimated as the percentage deviation of the hybrid mean relative to the best-performing parent (*BP*):

(4) $$H_{b}(\%)=(\frac{F_{1}-BP}{BP})\times 100$$

where *F*_1_ represents the mean performance of the hybrid, *P*_1_ and *P*_2_ denote the means of the parental lines, and *BP* corresponds to the superior parent. Positive estimates indicate hybrid superiority over the mid-parent (heterosis) or best parent (heterobeltiosis), whereas negative values indicate inferior hybrid performance.

#### 2.5.3 Single model

In order to compare the phenotypic performance of all evaluated genotypes, a one-way analysis of variance (ANOVA) was performed for all evaluated characteristics, including the genotype of the commercial reference cultivar, in order to evaluate the performance of the hybrids obtained from the diallel crosses described in Equation 1. The F-test, at a 5% significance level, was applied to determine the significance of genotypic effects, as described in Equation 5:

(5) $$Y_{ij}=\mu +g_{i}+\beta_{j}+\epsilon_{ij}$$

where $Y_{ij}$ is the phenotypic value of the ijth observation for the *i*th genotype in the *j*th block; μ is the overall mean; $g_{i}$ is the random effect of the *i*th genotype, with $g_{i}∼N(0,\sigma_{g}^{2})$; $\beta_{j}$ is the random effect of the *j*th block, with $\beta_{j}∼N(0,\sigma_{\beta }^{2})$; and $\epsilon_{ij}∼N(0,\sigma^{2})$ is the experimental error, assumed to be normally distributed with constant variance.

For mean comparisons, Dunnett’s test (Dunnett, 1955) was applied at a 5% significance level to detect differences between the means of parental lines and hybrids relative to the control. Each treatment *i* was compared to the check (C1) using the following test statistic 6:

(6) $$t_{i}=\frac{{\bar{Y}}_{i}-{\bar{Y}}_{0}}{\sqrt{MSE(\frac{1}{n_{i}}+\frac{1}{n_{0}})}}$$

where ${\bar{Y}}_{i}$ and ${\bar{Y}}_{0}$ are the means of treatment *i* and the control, respectively; *MSE* is the mean square error from Equation 5; and *n_i_* and *n*_0_ are the number of replications for treatment *i* and the control, respectively. The experimental coefficient of variation (*CV_r_*) was computed using Equation 7:

(7) $$CV_{r}=\frac{\sqrt{{\hat{\sigma }}_{e}^{2}}}{\hat{\mu }}\times 100$$

where ${\hat{\sigma }}_{e}^{2}$ is the residual variance and $\hat{\mu }$ is the overall mean for each trait across treatments.

Broad-sense heritability (*H*^2^) was estimated according to Equation 8:

(8) $$H^{2}=\frac{{\hat{\sigma }}_{g}^{2}}{{\hat{\sigma }}_{p}^{2}}$$

where ${\hat{\sigma }}_{g}^{2}$ is the genetic variance and ${\hat{\sigma }}_{p}^{2}$ is the phenotypic variance.

### 2.6 Multi-trait Genotype-Ideotype Distance Index

The Multi-trait Genotype-Ideotype Distance Index (MGIDI) was used to rank genotypes based on their proximity to an ideotype, considering the desired direction of each trait (Olivoto and Nardino, 2021). Initially, a factor analysis was performed on the rescaled data using the correlation matrix to account for trait correlations and reduce dimensionality. Factors with eigenvalues greater than one were retained according to the Guttman-Kaiser criterion (Yeomans and Golder, 1982), and a varimax rotation (Kaiser, 1958) was applied to obtain the final factor loadings. Genotypic scores were computed based on the rotated loadings and standardized means, and were subsequently used to calculate the MGIDI as:

(9) $$MGIDI_{i}=\;{\left[\sum_{j=1}^{f}{\left(\gamma_{ij}-\gamma_{j}\right)}^{2}\right]}^{0.5}$$

where *MGIDI_i_* is the index for genotype *i*, *γ_ij_* is the score of genotype *i* on factor *j*, *f* is the number of retained factors, and *γ_j_* is the score of the ideotype on factor *j*.

We sought to select an ideotype characterized by a plant architecture with shorter internodes of larger diameter, broader leaves, and larger fruits. Additionally, desirable features included low-acid fruits with high soluble solids content (Brix), as well as higher numbers of fruits per plant, yield per plant, and estimated productivity per hectare. The ideotype is defined as a hypothetical genotype with rescaled values equal to 100 for all traits. By default, all traits contribute equally to the index. However, differential weights can be assigned through a weight vector *θ_j_*, allowing prioritization of traits with greater agronomic relevance, such as NFP, YPP, and EYLD. The rescaling procedure ensures that traits for which lower values are desirable, such as FLC and IND, are correctly considered in the analysis. To identify the strengths and weaknesses of each genotype, the proportional contribution of each factor to the total MGIDI value (Olivoto et al., 2022) was computed as:

(10) $$\omega_{ij}=\frac{D_{ij}^{2}}{\sum_{j=1}^{f}D_{ij}^{2}}$$

where *D_ij_* is the squared distance between genotype *i* and the ideotype on factor *j*. Lower values of *ω_ij_* indicate that the genotype is closer to the ideal for the traits associated with that factor. All statistical analyses were performed using the metan package (Olivoto and Lúcio, 2020).

## 3 Results

### 3.1 GCA and SCA considering the NCII design

Combinatorial analysis based on the NCII design revealed no significant differences for most plant architecture traits between both parental groups, except for internode diameter (ID), which was significant in the GCA of the female parent group. In contrast, the interaction between parental groups showed significant effects (p *<* 0.01) for most traits, indicating the predominance of non-additive genetic effects associated with SCA (**Table 1**). For fruit quality traits, fruit size (FS) was significant in the female GCA (5%) and in the SCA interaction (1%), whereas pericarp firmness (PF) showed significant effects in both parental GCAs (5% and 1%), but not in the SCA. Regarding yield components, significant differences (5% and 1%) were observed in the GCA of both parental groups, while SCA effects were significant only for yield per plant (YPP). The number of fruits per plant (NFP) was significant for both parental GCAs (5% and 1%, respectively) but not for SCA, and yield per plant (YPP) was significant for male GCA (5%) and for SCA (1%), whereas estimated yield (EYLD) did not show significant differences. Trait means ranged from 1.30 to 77.50, indicating substantial variation among treatments, and the coefficients of variation were low to moderate, reflecting good experimental precision (**Table 1**).

**Table 1:** Summary of mean square estimates for GCA and SCA based on the NCII design.

Source of variation	df	Plant architecture				Fruit quality			Yield components		
		LL	LW	IL	ID	FS	PF	SSC/TA	NFP	YPP	EYLD
Block	2	0.43	0.59	7.61	0.20	0.0106	32.01	5.78	348.33	31.33	496.76
GCA (Male)	3	29.85ns	146.23ns	55.13ns	0.78ns	0.0099ns	52.76*	9.48ns	988.87*	88.99*	417.51ns
GCA (Female)	4	54.73ns	114.36ns	18.77ns	3.61*	0.1262*	17.29**	11.16ns	417.17**	37.52ns	193.37ns
SCA (Male × Female)	12	34.70*	79.37*	32.28*	0.87**	0.0173*	5.30ns	7.30ns	270.78ns	24.36**	342.73ns
Residual	38	1.13	0.94	12.44	0.33	0.0095	8.86	4.16	151.14	13.63	124.11
Mean		37.06	32.84	25.96	4.45	1.30	9.31	10.05	77.50	23.25	58.53
CV (%)		2.87	2.96	13.58	12.94	7.43	31.98	20.30	15.88	15.87	19.03

df, degrees of freedom; GCA, general combining ability; SCA, specific combining ability; LL, leaf length (cm); LW, leaf width (cm); IL, internode length (cm); ID, internode diameter (mm); FS, fruit size (g); PF, pericarp firmness (N); SSC/TA, soluble solids content to titratable acidity ratio (°Brix/% citric acid); NFP, number of fruits per plant (fruits plant^−1^); YPP, yield per plant (kg plant^−1^); EYLD, estimated yield (t ha^−1^). Significant at 1% (**); 5% (*); ns, not significant (F-test).

For plant architecture traits, P144 showed the best GCA for LL in both parental groups, P241 and P255 were the best combiners for LW, P230 showed the highest GCA for IL in both parental groups, and P144 and P255 were the best combiners for ID among male and female parents, respectively (**Table 2**). The highest SCA estimates were observed for H19 for LL, H4 for LW, H15 for IL, and H11 for ID. For fruit quality traits, the best GCA estimates were observed for P156 and P174 for FS, P121 and P255 for PF, and P241 and P230 for SSC/TA, considering male and female parents, respectively. The hybrids with the highest SCA were H5 for FS, H1 for PF, and H10 for SSC/TA. For yield-related traits, P144 showed the highest GCA for NFP and YPP among male parents, whereas P390 was the best female combiner for both traits. For EYLD, the highest GCA estimates were observed for P121 and P255 among male and female parents, respectively. Hybrid H9 showed the highest SCA for NFP, YPP, and EYLD. To support the interpretation of combining ability, the best male and female combiners based on GCA, as well as the hybrid combinations with the highest SCA for each trait, are presented in **Supplementary Table 2**.

**Table 2:** Superior parental lines and hybrids identified based on general and specific combining ability (GCA and SCA) for the evaluated traits.

Variable	Best male parent		Best female parent		Best hybrid			
	Male parent	Greater GCA	Female parent	Greater GCA	Male	Female	Hybrid	Greater SCA
Plant architecture								
LL	P144	-2.31150	P144	-2.63530	P121	P255	H19	-5.81150
LW	P241	3.9610	P255	3.1435	P144	P255	H4	7.0065
IL	P230	-2.87150	P230	-1.05050	P241	P230	H15	-4.27950
ID	P144	0.2155	P255	0.7185	P241	P174	H11	0.8745
Fruit quality								
FS	P156	0.0295	P174	0.1785	P144	P230	H5	0.1030
PF	P121	2.1030	P255	1.5135	P144	P174	H1	1.7740
SSC/TA	P241	0.7450	P230	1.0745	P156	P230	H10	3.9695
Yield components								
NFP	P144	11.2025	P390	7.6820	P156	P255	H9	12.5725
YPP	P144	3.3860	P390	2.1980	P156	P255	H9	3.8485
EYLD	P121	6.7575	P255	6.5650	P156	P255	H9	17.3190

GCA, general combining ability; SCA, specific combining ability; LL, leaf length (cm); LW, leaf width (cm); IL, internode length (cm); ID, internode diameter (mm); FS, fruit size (g); PF, pericarp firmness (N); SSC/TA, soluble solids content to titratable acidity ratio (°Brix/% citric acid); NFP, number of fruits per plant (fruits plant^−1^); YPP, yield per plant (kg plant^−1^); EYLD, estimated yield (t ha^−1^).

The variance decomposition revealed marked differences among traits regarding both the magnitude and the nature of genetic effects (**Table 3**). For plant architecture traits, dominance variance predominated, indicating a clear advantage for hybrid development and the exploitation of non-additive genetic effects. Within this group, ID exhibited relatively high broad-sense heritability, suggesting reliable selection and greater genetic control. Fruit quality traits were characterized by very low variance estimates; however, they showed high heritability values, indicating stable genetic control despite limited phenotypic variation. For yield components, dominance variance was predominant for most traits, while heritability values were generally moderate to low, reflecting the combined influence of heterosis and strong environmental effects.

**Table 3:** Genetic variance components for traits evaluated under the North Carolina Design II.

Variance component	Plant architecture				Fruit quality			Yield components		
	LL	LW	IL	ID	FS	PF	SSC/TA	NFP	YPP	EYLD
Genotypic variance	11.19	26.14	6.62	0.18	0.0026	-1.19	1.05	39.74	3.58	72.87
Additive variance	2.69	14.75	0.79	0.44	0.0172	8.33	0.93	120.14	10.81	-14.92
Dominance variance	44.76	104.56	26.46	0.73	0.0105	-4.75	4.18	158.99	14.31	291.50
h² (%)	19.69	42.79	4.08	60.42	82.95	70.30	16.45	47.80	47.80	-7.87

LL, leaf length (cm); LW, leaf width (cm); IL, internode length (cm); ID, internode diameter (mm); FS, fruit size (g); PF, pericarp firmness (N); SSC/TA, soluble solids content to titratable acidity ratio (°Brix/% citric acid); NFP, number of fruits per plant (fruits plant^−1^); YPP, yield per plant (kg plant^−1^); EYLD, estimated yield (t ha^−1^).

Overall, ID and FS exhibited the greatest potential for direct selection, whereas plant architecture and yield traits were more strongly influenced by dominance effects, supporting the adoption of breeding strategies focused on hybrid exploitation.

Mid-parent heterosis varied markedly among hybrids and traits (**Table 4**). Significant heterosis for LL was detected in H4, H6, H9, H12, H14, H18, and H19, with the highest positive estimate observed in H14 (23.13%). For LW, positive and significant heterosis was identified in H4, H6, H9, H11, H12, H13, H16, and H17, whereas significant negative heterosis occurred in H1, H8, H19, and H20. IL exhibited significant positive heterosis only in H7 (22.85%), while ID and FS showed limited significant responses, with significance observed only for FS in H6. Regarding yield-related traits, no hybrid showed significant positive mid-parent heterosis for NFP, YPP, or EYLD. However, H14 displayed a significant reduction in NFP and YPP (−33.09% and −33.10%, respectively). Overall, LL and LW exhibited more frequent and higher-magnitude heterotic responses than yield components, suggesting a stronger contribution of non-additive genetic effects to morphological traits than to yield-related traits.

**Table 4:** Heterosis (*h*, %) of the 20 experimental hybrids (H1–H20) evaluated for ten agronomic and fruit quality traits.

Hybrid	LL	LW	IL	ID	FS	PF	SSC/TA	NFP	YPP	EYLD
H1	-15.83***	-25.93***	-11.74*^ns^*	-14.19*^ns^*	6.81*^ns^*	12.08*^ns^*	0.15*^ns^*	13.20*^ns^*	13.19*^ns^*	5.60*^ns^*
H2	-1.80*^ns^*	-16.52***	-7.81*^ns^*	7.76*^ns^*	-0.77*^ns^*	-25.52*^ns^*	1.70*^ns^*	6.44*^ns^*	6.44*^ns^*	-3.41*^ns^*
H3	4.25*^ns^*	-10.02***	-1.68*^ns^*	14.38*^ns^*	-7.51*^ns^*	-10.64*^ns^*	6.26*^ns^*	-1.45*^ns^*	-1.43*^ns^*	-1.09*^ns^*
H4	9.98***	20.92***	-7.59*^ns^*	15.27*^ns^*	-3.50*^ns^*	-9.58*^ns^*	-10.72*^ns^*	11.36*^ns^*	11.34*^ns^*	10.19*^ns^*
H5	-10.97***	3.37*^ns^*	2.24*^ns^*	-14.59*^ns^*	7.34*^ns^*	-27.85*^ns^*	-10.34*^ns^*	4.24*^ns^*	4.23*^ns^*	-25.30*^ns^*
H6	13.62***	21.51***	-15.40*^ns^*	-21.30*^ns^*	14.18*	-23.54*^ns^*	-10.70*^ns^*	-14.08*^ns^*	-14.07*^ns^*	-29.28*^ns^*
H7	-13.18***	-0.66*^ns^*	22.85*	13.90*^ns^*	-0.38*^ns^*	-4.27*^ns^*	-11.39*^ns^*	-0.06*^ns^*	-2.31*^ns^*	4.33*^ns^*
H8	-9.81***	-23.75***	-14.80*^ns^*	-17.01*^ns^*	0.78*^ns^*	-23.48*^ns^*	-2.57*^ns^*	-10.54*^ns^*	-10.51*^ns^*	-25.20*^ns^*
H9	8.37***	10.78***	5.35*^ns^*	7.30*^ns^*	0.00*^ns^*	18.00*^ns^*	-7.73*^ns^*	9.28*^ns^*	9.27*^ns^*	30.36*^ns^*
H10	0.89*^ns^*	-6.44*	4.20*^ns^*	-0.87*^ns^*	-10.69*^ns^*	-0.29*^ns^*	44.17**	-1.56*^ns^*	-1.55*^ns^*	-6.33*^ns^*
H11	1.61*^ns^*	10.73***	13.90*^ns^*	13.24*^ns^*	-2.88*^ns^*	2.04*^ns^*	3.08*^ns^*	12.44*^ns^*	12.43*^ns^*	-1.24*^ns^*
H12	7.41**	6.62**	-1.86*^ns^*	-14.70*^ns^*	3.47*^ns^*	3.31*^ns^*	10.49*^ns^*	-0.48*^ns^*	-0.45*^ns^*	11.19*^ns^*
H13	-0.33*^ns^*	10.02***	14.31*^ns^*	3.33*^ns^*	-2.38*^ns^*	9.23*^ns^*	13.20*^ns^*	2.70*^ns^*	2.74*^ns^*	9.13*^ns^*
H14	23.13***	3.05*^ns^*	-5.20*^ns^*	5.04*^ns^*	-0.78*^ns^*	1.04*^ns^*	-5.36*^ns^*	-33.09*	-33.10*	-13.28*^ns^*
H15	-5.41*	-2.20*^ns^*	-18.03*^ns^*	-11.49*^ns^*	0.00*^ns^*	10.10*^ns^*	-4.06*^ns^*	-5.76*^ns^*	-5.75*^ns^*	1.01*^ns^*
H16	1.80*^ns^*	7.06**	10.51*^ns^*	-5.55*^ns^*	7.97*^ns^*	17.55*^ns^*	7.03*^ns^*	-5.82*^ns^*	-5.83*^ns^*	22.44*^ns^*
H17	2.55*^ns^*	13.74***	1.31*^ns^*	1.08*^ns^*	-5.84*^ns^*	-17.34*^ns^*	-6.06*^ns^*	12.46*^ns^*	12.47*^ns^*	-11.86*^ns^*
H18	4.78*	1.71*^ns^*	3.53*^ns^*	7.99*^ns^*	-4.80*^ns^*	17.52*^ns^*	-4.67*^ns^*	13.05*^ns^*	13.04*^ns^*	6.29*^ns^*
H19	-6.37**	-15.90***	-0.51*^ns^*	2.46*^ns^*	-2.36*^ns^*	18.76*^ns^*	-7.13*^ns^*	-7.21*^ns^*	-7.20*^ns^*	-4.13*^ns^*
H20	1.19*^ns^*	-14.87***	3.44*^ns^*	1.76*^ns^*	-1.56*^ns^*	11.25*^ns^*	-11.32*^ns^*	-11.54*^ns^*	-11.54*^ns^*	15.37*^ns^*

Heterosis was calculated relative to the mean performance of the two parental lines (mid-parent). Values represent percentage deviation of the hybrid from the mid-parent value and are followed by the significance level obtained by Student’s *t*-test (ns = not significant; *p < 0.05; **p < 0.01; ***p < 0.001). Positive values indicate superior hybrid performance compared to the mid-parent, whereas negative values indicate inferior performance.

LL, leaf length (cm); LW, leaf width (cm); IL, internode length (cm); ID, internode diameter (mm); FS, fruit size (g); PF, pericarp firmness (kgf cm^−2^); SSC/TA, soluble solids content to titratable acidity ratio (°Brix/% citric acid); NFP, number of fruits per plant (fruits plant^−1^); YPP, yield per plant (kg plant^−1^); EYLD, estimated yield (t ha^−1^). Significance: ns, not significant; *p < 0.05; **p < 0.01; ***p < 0.001.

Heterobeltiosis (H*_b_*) showed a heterogeneous pattern across hybrids and traits (**Table 5**). For LL, significant positive H*_b_* was observed in H6 (13.27%) and H14 (20.14%), whereas significant negative estimates were detected in H1, H5, H7, H8, H15, and H19. For LW, significant positive HB occurred in H4, H6, H9, H11, and H17, while most other hybrids exhibited significant negative values, particularly H1, H8, H19, and H20. IL did not present significant heterobeltiosis in any hybrid. In contrast, ID showed significant negative HB in H1, H5, H6, and H8. FS exhibited a significant reduction only in H10. For PF, no hybrid surpassed the best parent significantly. Regarding fruit quality, SSC/TA ratio displayed significant positive heterobeltiosis only in H10 (41.06%). For yield-related traits, significant negative HB was detected in H14 for NFP and YPP (−33.12% and −33.13%, respectively), and in H6 for EYLD (−32.46%). No hybrid exhibited significant positive heterobeltiosis for yield components. Overall, heterobeltiosis was more frequently observed in vegetative traits than in yield-related traits, with limited evidence of hybrid superiority over the best parent for yield features.

**Table 5:** Heterobeltiosis (H*_b_*, %) of the 20 experimental hybrids (H1–H20) for agronomic and fruit quality traits.

Hybrid	LL	LW	IL	ID	FS	PF	SSC/TA	NFP	YPP	EYLD
H1	-18.38***	-32.30***	-15.88*^ns^*	-21.46*	0.68*^ns^*	-1.94*^ns^*	-2.00*^ns^*	7.71*^ns^*	7.70*^ns^*	3.02*^ns^*
H2	-3.26*^ns^*	-21.74***	-15.50*^ns^*	7.30*^ns^*	-1.53*^ns^*	-26.36*^ns^*	0.51*^ns^*	4.33*^ns^*	4.32*^ns^*	-5.67*^ns^*
H3	1.79*^ns^*	-10.32***	-7.00*^ns^*	14.38*^ns^*	-10.69*^ns^*	-19.67*^ns^*	0.56*^ns^*	-6.82*^ns^*	-6.80*^ns^*	-1.29*^ns^*
H4	2.80*^ns^*	9.78***	-10.68*^ns^*	9.69*^ns^*	-5.34*^ns^*	-24.12*^ns^*	-15.15*^ns^*	0.20*^ns^*	0.19*^ns^*	2.21*^ns^*
H5	-11.68***	2.83*^ns^*	-1.00*^ns^*	-21.46*	6.11*^ns^*	-35.18*^ns^*	-16.62*^ns^*	-5.43*^ns^*	-5.45*^ns^*	-25.92*^ns^*
H6	13.27***	18.00***	-16.22*^ns^*	-24.04*	8.78*^ns^*	-29.83*^ns^*	-13.43*^ns^*	-18.31*^ns^*	-18.31*^ns^*	-32.46*
H7	-14.30***	-0.86*^ns^*	19.04*^ns^*	8.23*^ns^*	-2.24*^ns^*	-8.19*^ns^*	-14.93*^ns^*	-7.67*^ns^*	-9.75*^ns^*	-0.26*^ns^*
H8	-10.18***	-28.14***	-14.97*^ns^*	-21.46*	-3.73*^ns^*	-27.72*^ns^*	-2.89*^ns^*	-14.41*^ns^*	-14.38*^ns^*	-26.96*^ns^*
H9	4.04*^ns^*	6.81**	2.82*^ns^*	-3.10*^ns^*	-2.99*^ns^*	3.60*^ns^*	-16.53*^ns^*	8.31*^ns^*	8.31*^ns^*	18.51*^ns^*
H10	-2.64*^ns^*	-11.68***	1.52*^ns^*	-3.85*^ns^*	-12.69*	-5.86*^ns^*	41.06**	-1.58*^ns^*	-1.57*^ns^*	-7.60*^ns^*
H11	0.23*^ns^*	8.70***	12.65*^ns^*	6.88*^ns^*	-8.78*^ns^*	-0.49*^ns^*	-0.74*^ns^*	5.97*^ns^*	5.95*^ns^*	-2.37*^ns^*
H12	4.30*^ns^*	1.90*^ns^*	-4.79*^ns^*	-17.10*^ns^*	3.08*^ns^*	-10.68*^ns^*	5.37*^ns^*	-8.84*^ns^*	-8.81*^ns^*	9.80*^ns^*
H13	-2.37*^ns^*	-0.82*^ns^*	13.94*^ns^*	0.00*^ns^*	-5.38*^ns^*	3.40*^ns^*	12.78*^ns^*	-2.61*^ns^*	-2.57*^ns^*	5.48*^ns^*
H14	20.14***	1.90*^ns^*	-7.60*^ns^*	-3.10*^ns^*	-2.31*^ns^*	-1.39*^ns^*	-14.91*^ns^*	-33.12*	-33.13*	-16.74*^ns^*
H15	-10.18***	-11.68***	-20.24*^ns^*	-16.06*^ns^*	-0.77*^ns^*	4.27*^ns^*	-5.48*^ns^*	-6.66*^ns^*	-6.65*^ns^*	-3.34*^ns^*
H16	1.49*^ns^*	1.55*^ns^*	6.19*^ns^*	-13.04*^ns^*	0.68*^ns^*	9.20*^ns^*	2.70*^ns^*	-6.94*^ns^*	-6.95*^ns^*	16.10*^ns^*
H17	0.61*^ns^*	10.79***	1.18*^ns^*	0.87*^ns^*	-6.20*^ns^*	-31.46*^ns^*	-8.98*^ns^*	7.85*^ns^*	7.87*^ns^*	-16.51*^ns^*
H18	3.72*^ns^*	-1.89*^ns^*	0.25*^ns^*	7.30*^ns^*	-7.03*^ns^*	6.13*^ns^*	-11.47*^ns^*	12.45*^ns^*	12.44*^ns^*	-1.36*^ns^*
H19	-9.59***	-20.79***	-5.73*^ns^*	-3.10*^ns^*	-3.13*^ns^*	15.69*^ns^*	-10.01*^ns^*	-11.53*^ns^*	-11.51*^ns^*	-4.27*^ns^*
H20	-2.93*^ns^*	-17.74***	-2.15*^ns^*	-5.87*^ns^*	-1.56*^ns^*	0.53*^ns^*	-19.05*^ns^*	-14.91*^ns^*	-14.92*^ns^*	6.05*^ns^*

HB was computed as the percentage deviation of each hybrid relative to its superior parent (best-parent performance). Reported values correspond to the proportional gain or reduction of the hybrid compared to the best parent and are followed by the significance level determined by Student’s *t*-test (ns, not significant; *p < 0.05; **p < 0.01; ***p < 0.001). Positive estimates indicate hybrid superiority over the best-performing parent, whereas negative estimates indicate performance below the parental benchmark.

LL, leaf length (cm); LW, leaf width (cm); IL, internode length (cm); ID, internode diameter (mm); FS, fruit size (g); PF, pericarp firmness (kgf cm^−2^);.

SSC/TA, soluble solids content to titratable acidity ratio (°Brix/% citric acid); NFP, number of fruits per plant (fruits plant^−1^); YPP, yield per plant (kg. plant^−1^); EYLD, estimated yield (t ha^−1^). Significance: ns, not significant; *p < 0.05; **p < 0.01; ***p < 0.001.

### 3.2 Hybrid evaluation

Analysis of variance revealed significant differences (*p<*0.01) among treatments for all plant architecture traits, indicating the presence of substantial genetic variability. These differences among means also confirmed the distinctiveness between groups. The coefficients of variation (CV) express low and medium values, with the highest CV being for LW (17.85%) (**Table 6**). For fruit quality traits, analysis of variance showed that PF was significant at the 5% level (*p<*0.05), pH was not significant, and the SSC/TA ratio was significant at the 1% level (*p<*0.01). The range of means, minimum, and maximum values indicated differences between groups. However, for pH, the similarity among mean, minimum, and maximum values within groups supports the non-significance found in the ANOVA, confirming low variability for this trait. Regarding CV, pH showed a low value, while SSC/TA showed a moderate CV, and PF showed a very high CV. For yield-related traits, significant differences were observed at the 1% level (*p<*0.01) by the F-test. Means and the range between minimum and maximum values were not highly variable. For CV, NFP, YPP, and EYLD exhibited high values, and PF had a very high CV (**Table 6**).

**Table 6:** Mean squares from ANOVA for plant architecture, fruit quality, and yield-related traits in tomato genotypes.

SV	df	Plant architecture				Fruit quality			Yield components		
		LL	LW	IL	ID	FS	PF	SSC/TA	NFP	YPP	EYLD
Block	2	1.63	11.41	4.23	0.08	0.008	18.38	1.05	64.78	0.44	147.15
Genotype	29	43.83**	109.64**	35.80**	1.91**	0.064**	16.38*	13.09**	859.73**	3.11**	846.47**
Residual	58	9.33	29.74	12.54	0.27	0.008	9.16	3.55	268.60	0.72	197.60
Mean		35.33	30.82	25.46	4.31	1.33	8.66	10.35	70.30	3.09	51.33
Minimum		22.00	12.00	12.00	2.00	0.96	4.00	7.50	22.67	0.58	9.59
Maximum		49.00	49.00	34.00	6.00	1.86	17.56	24.17	124.67	6.04	100.74
*H*^2^ (%)		79.30	73.28	64.29	85.85	87.03	43.66	73.17	67.75	75.48	75.17
CV (%)		8.64	17.76	13.90	12.24	6.90	34.14	18.21	23.31	27.45	27.38

SV, Source of variation; df, degrees of freedom; LL, leaf length (cm); LW, leaf width (cm); IL, internode length (cm); ID, internode diameter (mm); FS, fruit size (g); PF, pericarp firmness (kgf cm^−2^); SSC/TA, soluble solids content to titratable acidity ratio (°Brix/% citric acid); NFP, number of fruits per plant (fruits plant^−1^); YPP, yield per plant (kg plant^−1^); EYLD, estimated yield (t ha^−1^). Significance levels: ** significant at 1%; * significant at 5%; ns: non-significant according to the F-test.

Significant differences among genotypes were observed for most traits related to plant architecture, fruit quality, and yield components (**Table 6**). All plant architecture traits (LL, LW, IL, ID) showed significant variation (p *<* 0.01), with heritability ranging from 64.29% (IL) to 85.85% (ID). For fruit quality, PF and SSC/TA were significant (p *<* 0.05 and p *<* 0.01, respectively), while pH was not. Heritability values were moderate for PF (43.66%) and high for SSC/TA (73.17%). All yield-related traits (FS, NFP, YPP, EYLD) were highly significant (p *<* 0.01), with heritability ranging from 67.75% (NFP) to 87.03% (FS). Coefficients of variation were acceptable, confirming the precision of the experiment.

**Figure 1:**
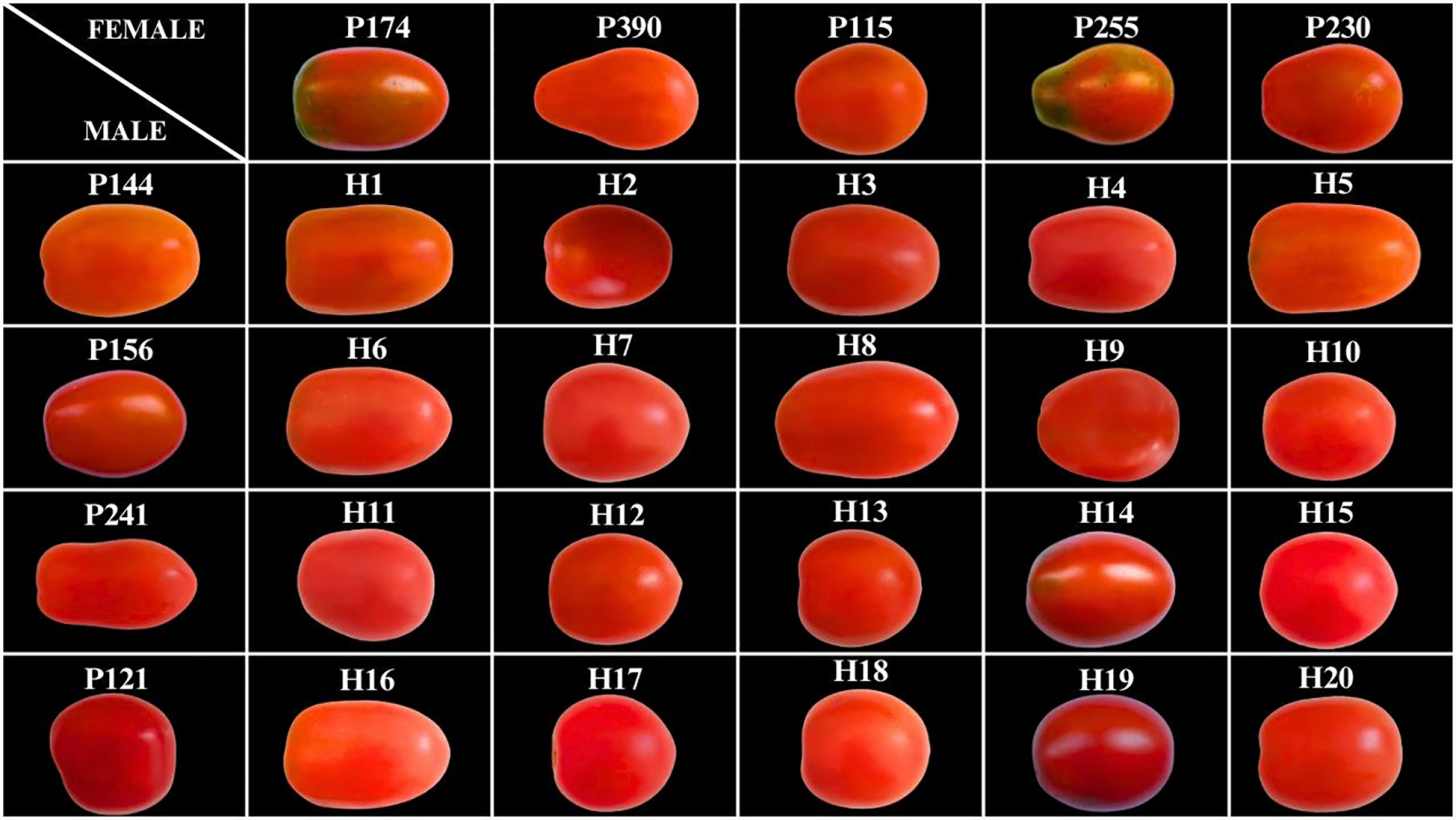
Visual representation of the parental lines evaluated in the study, highlighting the morphological diversity between the hybrids and their respective parents. Genotypes P144 to P121, arranged in the first column, represent the male parents, while genotypes P174 to P230, positioned in the first row, comprise the female parents. Genotypes labeled with the prefix “H” (e.g., H1 to H20) represent the hybrids derived from the crosses.

**Figure 1** illustrates the morphology of the parental lines and the 20 individuals comprising the progeny. Regarding fruit size, hybrids H2, H3, H5, and H10, as well as inbred lines P121 and P115, were classified as small. Hybrids H14, H16, and H19, along with line P230 and the control, were classified as large. The remaining genotypes were categorized as medium-sized fruits. For the presence of green shoulder, only hybrids 9 and 14 and inbred lines P174 and P255 exhibited this trait. Fruit classification followed the guidelines of the Brazilian Ministry of Agriculture, Livestock, and Food Supply (MAPA) (Brazilian Ministry of Agriculture, Livestock and Food Supply, 2018), fruits with a transverse diameter greater than 60mm are considered large; those greater than 50mm and up to 60mm are considered medium; and those greater than 40mm and up to 50 mm are considered small. In terms of fruit color at full maturity, a range from orange to deep red was observed, with red being the predominant color.

**Figure 2:**
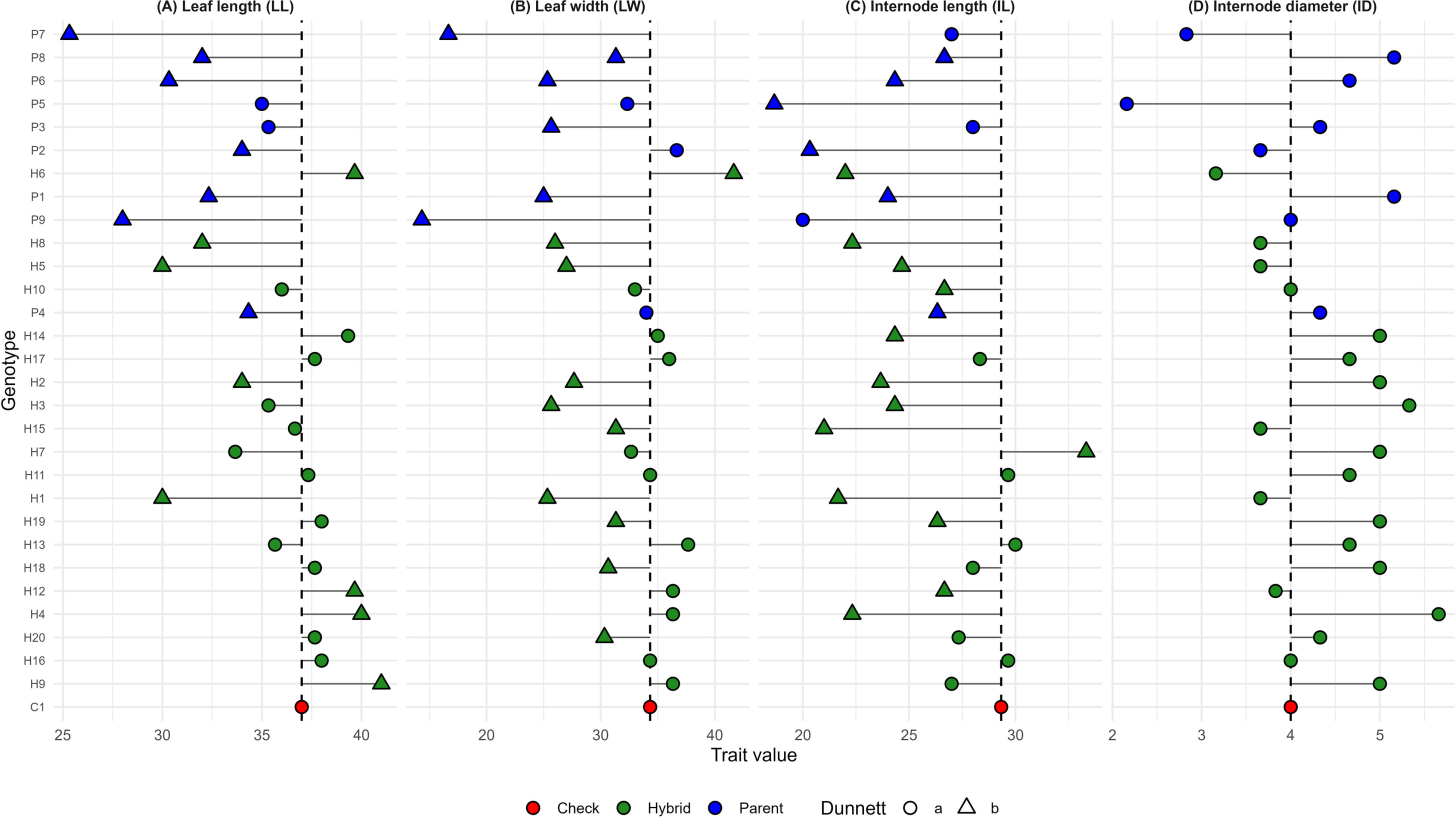
Dunnett’s test for **(A)** leaf length (LL), **(B)** leaf width (LW), **(C)** internode length (IL), and **(D)** internode diameter (ID). The dashed vertical line represents the control mean, allowing the identification of genotypes with means higher or lower than the control. Filled circles indicate genotypes not significantly different from the control (a), whereas filled triangles indicate significant differences according to Dunnett’s test (b). Red indicates the check, green indicates hybrids, and blue indicates parental lines.

**Figure 3:**
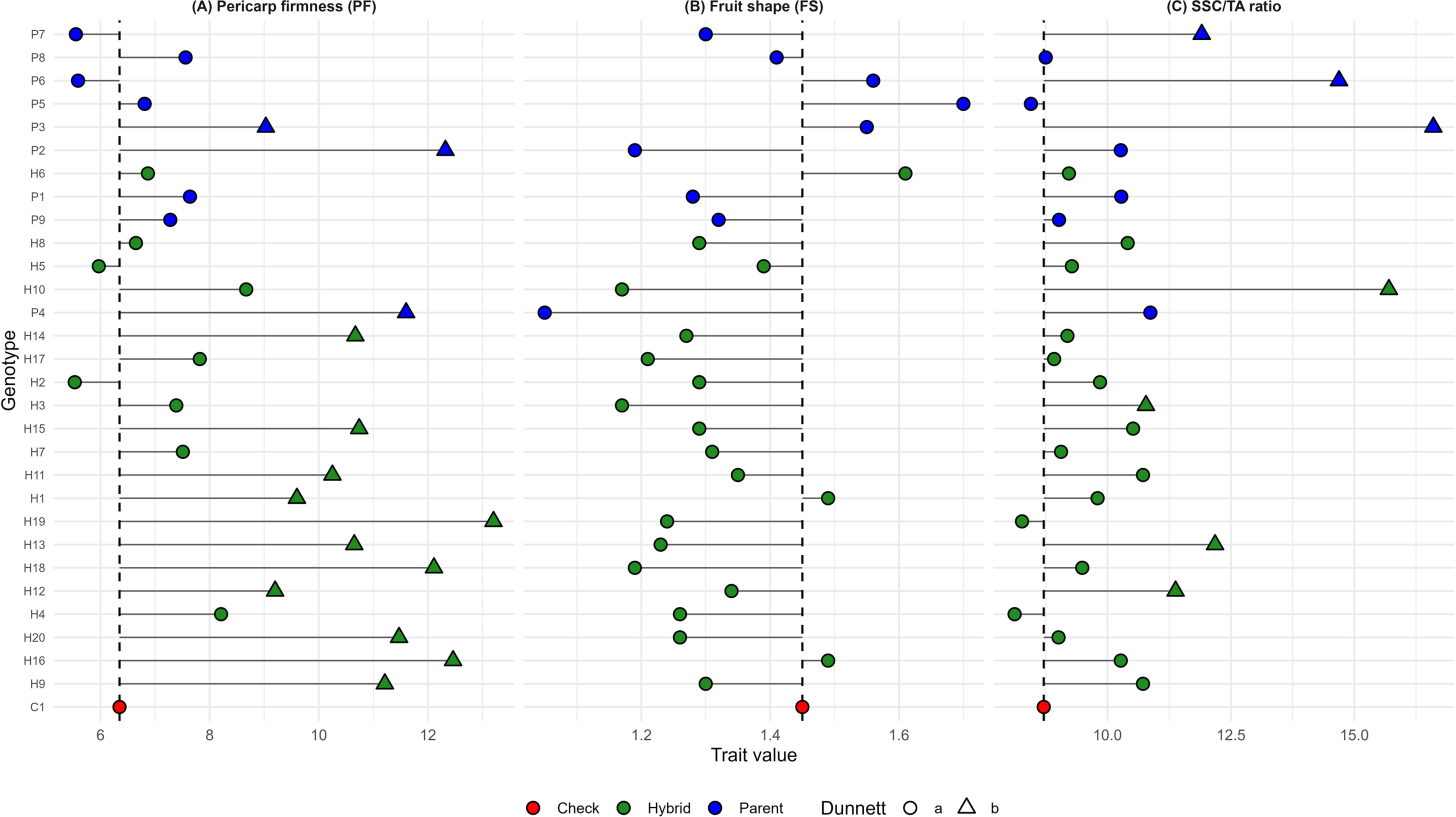
Dunnett’s test for **(A)** pericarp firmness (PF), **(B)** fruit shape (FS), and **(C)** soluble solids to titratable acidity ratio (SSC TA). The dashed vertical line represents the control mean, allowing the identification of genotypes with means higher or lower than the control. Filled circles indicate genotypes not significantly different from the control (a), whereas filled triangles indicate significant differences according to Dunnett’s test (b). Red indicates the check, green indicates hybrids, and blue indicates parental lines.

**Figure 4:**
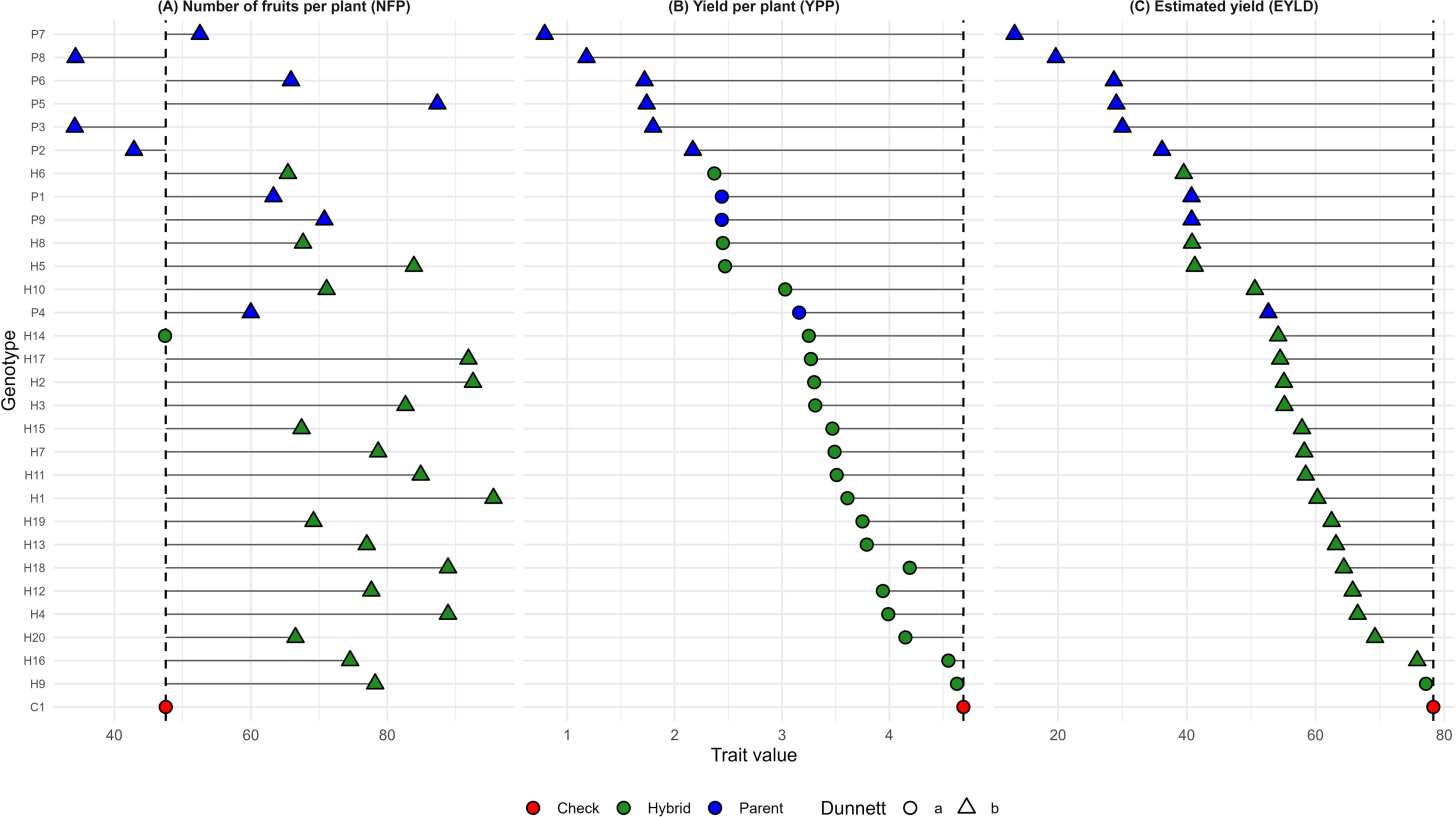
Dunnett’s test for **(A)** number of fruits per plant (NFP), **(B)** yield per plant (YPP), and **(C)** estimated yield (EYLD). The dashed vertical line represents the control mean, allowing the identification of genotypes with means higher or lower than the control. Filled circles indicate genotypes not significantly different from the control (a), whereas filled triangles indicate significant differences according to Dunnett’s test (b). Red indicates the check, green indicates hybrids, and blue indicates parental lines.

Dunnett’s test was used to compare the performance of the hybrids with the control. The treatment means were compared exclusively with the control mean. In the graphs, green represents the hybrids (H1 to H20), blue represents the parental lines (P144, P156, P241, P121, P174, P390, P115, P255 and P230), and red represents the control (C1). The circular figure “a” indicates that the treatment mean does not differ from that of the control, while the triangular figure “b” indicates a significant difference, which may be greater or lesser (**Figures 2**–**4**).

Regarding plant architecture characteristics, all hybrids presented average values equivalent to the control, with H6 standing out as having the highest average in leaf diameter (LW) and H1, H2, H5, and H8 having the lowest averages, indicating genotypes with shorter leaf length (IL) (**Figure 2**). Regarding fruit quality, most hybrids showed superior performance to the control for PF, while no difference was observed for FS. In the SSC/TA ratio, most hybrids showed equal performance compared to the control, indicating low acidity (**Figure 3**). As for yield traits, most hybrids presented averages similar to the control, with 19 hybrids exhibiting higher averages for NFP and hybrid 9 showing an average equal to the control for EYLD (**Figure 4**).

**Figure 5:**
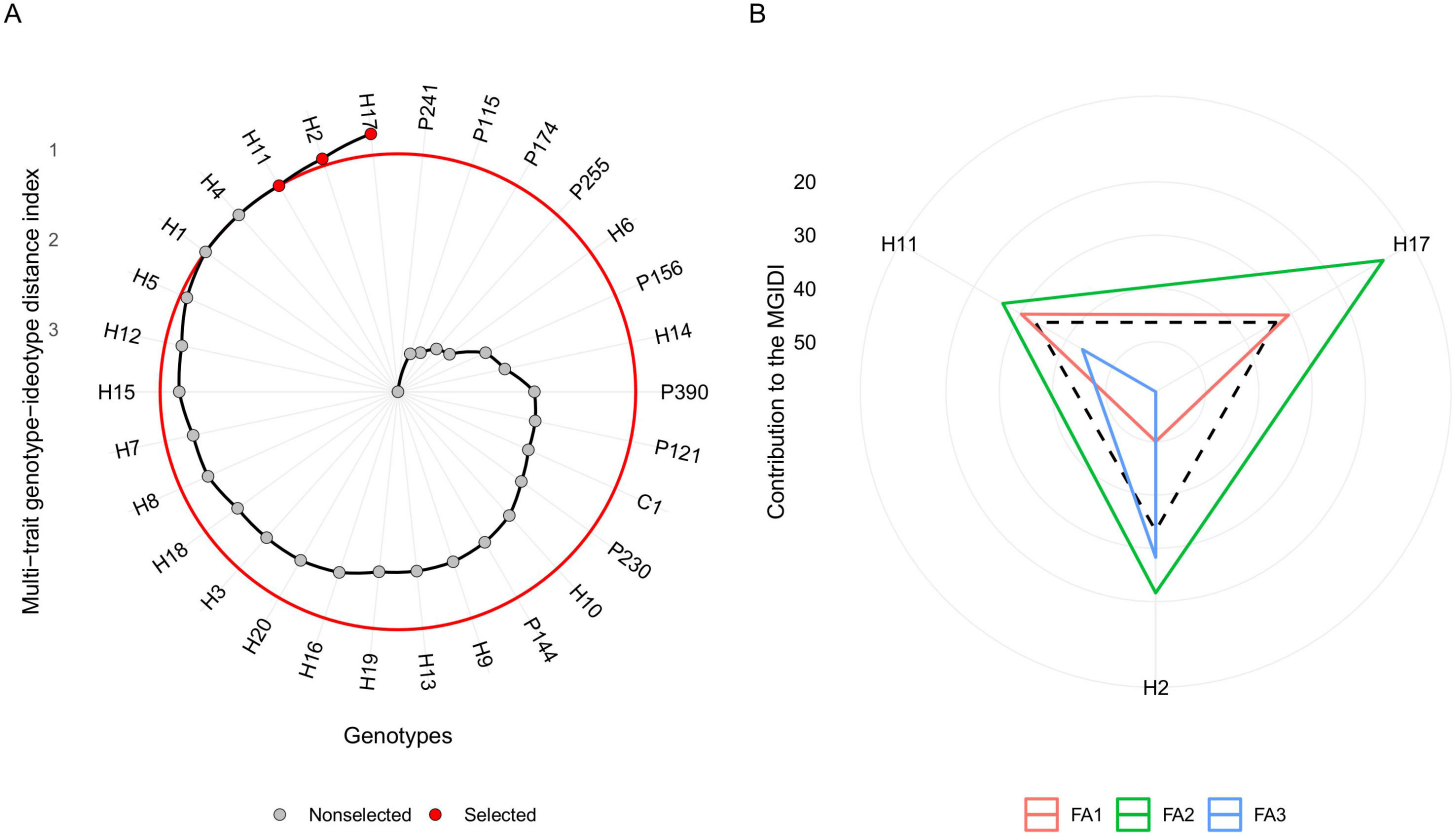
**(A)** Ranking of genotypes in ascending order based on the multi-trait stability index (MGIDI). Genotypes selected by the MGIDI are highlighted in red. **(B)** Strengths and weaknesses of the selected hybrids, showing the proportion contributed by each factor to the overall MGIDI score.

Simultaneous selection based on the MGIDI index allowed the identification of hybrids H17, H2, and H11 as the genotypes with superior agronomic performance, considering, in an integrated way, plant architecture (FA1), fruit quality (FA2), and productivity characteristics (FA3) (**Figure 5A**). Following Kayser’s assumptions (eigenvalues *>* 1), three factors were obtained, and this retention was consistent with the multivariate organization of the dataset, as the characteristics were distributed in distinct correlation groups, indicating different patterns of variation. Thus, H17 was the closest to the ideotype, demonstrating a high balance among the factors, although FA2 represents the main limitation. Genotype H11 showed a greater contribution from factorial factor FA1 to the distance from the ideotype, suggesting lower performance in this set of traits. In contrast, H2 showed a greater contribution from FA3, indicating that its limitations are concentrated in the variables grouped in this factor (**Figure 5B**).

## 4 Discussion

### 4.1 Genetic variability among parents and hybrids

The significant differences observed for most plant architecture traits, fruit quality attributes, and yield components among the evaluated genotypes indicate the presence of substantial genetic variability within the germplasm set. Such variability constitutes a fundamental prerequisite for achieving genetic gain in breeding programs, particularly in tomato, where hybrid development remains the primary strategy for cultivar release. Broad genetic divergence among parental lines enhances the probability of identifying superior combinations, increases the efficiency of selection, and supports the exploitation of heterosis. These findings are consistent with the results reported by Rasheed et al. (2023), who emphasized that adequate levels of genetic variability are essential for the development of high-yielding genotypes, as limited productivity is often associated with a narrow genetic base.

The wide range of variation observed in the evaluated traits indicates the presence of contrasting allelic combinations among the parental lines. Although the lines originated from the same breeding program, prior selection based on the number of resistance alleles and recombination events over generations led to the expression of relevant quantitative differences. It is known that in polygenic traits, even with similar genetic bases, a wide phenotypic divergence can result when there is complementarity of favorable alleles. Zannat et al. (2023) report the importance of verifying the existence of genetic variability in gene banks, as this can lead to the discovery of superior genotypes with higher yield, adaptability to diverse locations, and resistance to diseases, such as tomato yellow leaf roll virus (TYLCV). In the present work, there are parental lines that confer resistance to this virus, and as observed by the cited authors, these accessions are essential for establishing a new breeding population.

Narrow-sense heritability estimates were moderate to high for most traits, with ID (60.42%), FS (82.95%), and PF (70.30%) standing out. High heritability values indicate that much of the phenotypic variance is due to additive genetic effects, which increases selection efficiency. For the aforementioned traits, high heritability suggests that direct phenotypic selection can result in rapid and efficient genetic gains over generations, while yield traits show moderate heritability, inferring that their quantitative control is complex and has a greater environmental influence. Similar results were found by Singh et al. (2025), who had narrow-sense heritabilities above 60% for fruit quality traits and low heritabilities for yield components.

The superiority of several hybrids in relation to the parents identified by heterosis and heterobeltiosis high-lights the potential of the evaluated lines, mainly regarding plant architecture. The superior performance of the hybrids in relation to the parents suggests the complementarity of superior alleles, which is particularly relevant in tomato breeding, where hybrid vigor is widely exploited to increase yield (Dufera, 2025). For the attributes that did not show significant heterosis and heterobeltiosis (ID, PF, SSC/TA, NFP, YPP, and EYLD), in practical terms, this means that the combination between the lines for these characteristics did not result in hybrid vigor. Fruit quality characteristics in tomato plants frequently show greater additive control, which is reinforced by the non-significance of SCA (Ene et al., 2023). The purpose of this type of research is precisely to evaluate the combining ability of parental lines, since the success of heterosis depends heavily on the selection of suitable parents that complement each other and improve the vigor of important agronomic traits. The initial combination of genotypes may not result in hybrids with high vigor, but once the GCA shows significant, high, and positive estimates, those parents with higher averages can be recombined with other parents or destined for recurrent selection, considering that there is a predominance of additive effect and heterosis is not advantageous for the initial cross (Rabiei and Akhlagh, 2023).

Analysis of variance indicated significant effects for the treatment factor (*p <* 0.05 or *p <* 0.01), confirming genetic variability among the evaluated hybrids. This variability, attributed to the genetic differences among parental lines, highlights the potential for selecting superior genotypes for key agronomic and quality traits, as noted by Nguyen et al. (2024) and Oladokun et al. (2022). Broad-sense heritability values for most traits indicate the need for multi-environment trials to enhance selection accuracy, in line with findings by Zorb et al. (2020). Most coefficients of variation (CV) were classified as low or moderate (**Table 6**), demonstrating satisfactory experimental precision and genotype uniformity (Glogovac et al., 2022a). Higher CVs for yield-related traits may reflect environmental heterogeneity and experimental design factors (Pélabon et al., 2020).

The selection of the best parents should also be associated with the morphological and sensory attributes of the fruits, such as color, flavor, appearance, and shelf life, which are crucial for consumer acceptance (Zheng et al., 2023). Fruits with green shoulders, regulated by the *SIBL2* gene, showed less commercial appeal, while fruits with intense red coloration were preferred (Niu et al., 2022). Therefore, genetic improvement strategies should integrate productive and quality characteristics, especially if the variety is intended for fresh consumption, which requires greater morphological qualities in product development, as demonstrated by Ortega-Salazar et al. (2025), who differentiated lines intended for processing from lines intended for the fresh market. Dunnett’s test enabled the comparison of all hybrids with the control. For most traits, hybrids performed as well as or better than the control, with 19 hybrids showing a superior number of fruits per plant (NFP). Similar results have been reported in tomato by Peixoto et al. (2020) and Oliveira et al. (2021). These findings underscore the potential of the studied hybrids for future breeding efforts.

### 4.2 Combining ability and genetic control of traits

Considering that GCA is a measure of additive effect, when crossing a lineage with several others, we will have the average performance of this inbred line in all its crosses, and therefore, the expression of the deviation from the mean of the combinations was considered significant for the ID variable in the plant architecture category (**Table 1**). This observation implies that additive genetic variance is predominant for this trait, considering that the additive component refers to the average capacity of the inbred lines individuals involved in the cross (Datta et al., 2021; Orellana et al., 2024). The other variables are under the domain of dominance effects and specific interactions between male and female, since SCA is significant in all of them (**Table 1**). This result shows the strong influence of non-additive gene effects, such as dominance and possible epistatic interactions. In this case, exploiting heterosis is the most efficient strategy to maximize genetic gains in these traits, since such effects are not fixable by selection in inbred lines (Shrivastav et al., 2025).

Plant architecture is a classic target in breeding programs aimed at increasing productivity and optimizing plant arrangement in cultivated areas. Genetic-statistical methods, such as diallel analysis, assist in selecting the most promising parents for crosses (Santos et al., 2025). The appropriate choice of parents is essential, as traits like internode length and diameter influence plant support and compactness, which are desirable for both commercial cultivation and fresh market consumption (Liu et al., 2021; Naeem et al., 2020). In this study, the ID variable shows the greatest contribution of additive variance and high heritability. Therefore, this trait is suitable for recurrent selection or methods based on pure lines. The significant effect of GCA indicates the existence of parents that contribute favorable alleles, making them strategic for continuous use in the program. The correlation between additive variance and heritability is demonstrated by Mahmoud et al. (2023) when selecting tomato lines tolerant to tomato yellow leaf roll virus (TYLCD) based on estimates of heritability and genotypic variance. Parents with higher, especially positive, GCA values tend to contribute significantly to the improvement of the evaluated traits and are preferred in breeding programs (Ezin et al., 2023). Negative values indicate potential to reduce the respective trait. According to Cruz et al. (2014), effects close to zero suggest little difference from the diallel population mean, whereas extreme effects (positive or negative) highlight superior or inferior parents relative to others. Further corroborating Cruz et al. (2014), the choice of parents with the highest GCA indices is ideal for establishing new populations, as the selection of homozygous lines will be favored, in the case of self-pollinating plants such as tomatoes. For breeders, GCA carries greater importance due to the fact that it contributes to the increase in additive gene expression, since non-additive components refer to heterosis verified by the difference between the average of the evaluated characteristics in the hybrids and the average of that same trait in the parents.

The results indicate that there are additive effects in controlling the quality characteristics of the fruits, with the exception of the SST/TA variable, which did not show a significant effect. (**Table 1**). The lack of significance indicates that there are no real differences between the effects of GCA and SCA among the treatments for these traits. The fact that the variables are not significant may be due to the proximity of the genetic parameters between the inbred lines, which share the same gene pool. Fruit firmness, for example, is one of the most important interests in tomato breeding, since firm fruits reduce production loss, especially during transport. In addition, firmness confers quality, being an essential aspect for fruits consumed fresh (Xu et al., 2022). FS and PF exhibit significant GCA and high heritability, suggesting additive genetic control, which is favorable in the improvement of these traits (Adubi, 2025), which obtained high heritability estimates and considers phenotypic selection for these characters reliable and effective. Since fruit firmness is associated with cell wall composition and pectin metabolism (Du et al., 2025), additive control facilitates the achievement of cumulative gains and greater stability between generations.

The sensory traits of the fruit are important indicators of quality, and it is important to emphasize that these are influenced by the genotype, the environment, and the interaction between them (Felföldi et al., 2022). The predominance of additive effects highlighted in **Table 1** indicates the existence of parents that will contribute more to improving the characteristic, making it important to understand the estimates of GCA and SCA for the appropriate selection of parents to be used in hybridization. Previous studies report that the agreement between GCA results and heritability estimates strengthens the genetic interpretation, since traits with high additive variance and high heritability tend to respond efficiently to direct selection, while those with a predominance of dominance variance should be explored via hybrids (Reddy et al., 2023; Singh et al., 2025). In the present study, most traits showed GCA effects superior to those of SCA, which is especially relevant for the choice of parents in recurrent or self-pollinated selection programs. Finally, the presence of significant additive effects for half of the productivity traits demonstrates the potential of the studied parents to improve productivity indices, corroborating previous findings (Souza et al., 2012; Andrade et al., 2014). Thus, NCII analysis not only guides the selection of parents but also optimizes the development of superior hybrids and inbred lines for the target traits of the genetic improvement program (Santos et al., 2025).

The predominance of dominance variance for most traits indicates a strong influence of non-additive effects, suggesting the need to exploit heterosis for productivity gains. Variables that showed a greater contribution of additive variance and high heritability (ID, FS and PF) confirm the potential for direct selection and consistent gains between cycles (Raja et al., 2022). In contrast, variables with low heritability indicate a strong environmental influence. Negative estimates suggest high residual variance, which is common in highly quantitative and environment-dependent traits. Therefore, it is understood that integrated strategies combining direct selection for highly heritable traits with the use of hybrids for dominance-dependent traits tend to maximize genetic gain.

### 4.3 Multivariate selection and implications for tomato breeding

Although GCA and SCA analyses report parents with additive and non-additive effects of interest, these estimates do not capture possible trade-offs between different traits. Thus, the MGIDI index becomes relevant, as it is able to integrate, in a single metric, information on plant architecture, fruit quality, and yield components. As a complement to diallel analysis, MGIDI identifies genotypes closer to the ideotype based on multiple agronomic and quality traits (Olivoto et al., 2022). Hybrids H17, H2, and H11 were identified as the best performing, with H17 showing the best overall balance between factors, although it presents specific limitations in FA2 (fruit quality), indicating a need for adjustments in the characteristics grouped in this factor. H11 showed a greater contribution from FA1 (plant architecture), suggesting inferior performance in this group of variables, while H2 had a greater impact on FA3 (yield), revealing weaknesses concentrated in this set.

From a biological point of view, the use of multivariate indices is quite relevant in complex crops such as tomatoes (Akand et al., 2024), where agronomic and quality traits are frequently correlated. Selection based solely on yield attributes can compromise architectural and sensory attributes. Multivariate selection allows maintaining a functional balance between traits, increasing the probability of commercial acceptance. The MGIDI index demonstrated efficiency in multi-trait selection for various crops, increasing genetic gains in yield, quality, and adaptability (Debnath et al., 2024; Pallavi et al., 2024; Mohanty et al., 2025). Despite the promising results of the H17, H2, and H11 hybrids in the evaluated experimental environment, further validation in multi-environment trials considering the G×E interaction is necessary, since quantitative traits such as yield, number of fruits per plant, and firmness are strongly influenced by environmental variations. Additionally, incorporating genome-wide molecular markers and genomic prediction models may improve parental selection and enhance the prediction of hybrid performance for complex quantitative traits. The inclusion of multiple environments allows for estimating the magnitude of the G×E interaction, verifying the stability of the genotype ranking, and obtaining more accurate heritability estimates; only then is it recommended to indicate them as new cultivars (Tyagi et al., 2024).

## 5 Conclusions

The study revealed a wide genetic variability among the evaluated Italian tomato genotypes. GCA and SCA analysis indicated a greater predominance of non-additive effects for plant architecture and productivity traits, suggesting that the exploration of hybrids in tomato breeding is essential. However, for some traits (ID, FS, and PF), additive effects and high heritabilities predominated. The integration of the NCII model factor analysis with the MGIDI index allowed the identification of hybrids H17, H2, and H11 as the most promising, showing better multivariate balance between the traits studied simultaneously. H17 stood out due to the greater contribution of FA2, indicating that its selection was strongly influenced by productivity attributes. H2 showed a greater reinforcement from FA3, inferring that its selection was influenced by fruit quality attributes. H11, meanwhile, obtained a balance between the factors, indicating favorable simultaneous performance for plant architecture, fruit quality, and productivity. Therefore, it is recommended to verify the behavior and adaptability of these genotypes in different locations before making commercial recommendations.

## Data Availability

The original contributions presented in the study are included in the article/**Supplementary Material**, Further inquiries can be directed to the corresponding author.
