# Supplementary material for: Integrating combining ability and multivariate analysis for the selection of superior tomato hybrids
Source: Front Plant Sci. 2026 Apr 10;17:1788062. doi: 10.3389/fpls.2026.1788062 (PMC13106503; doi:10.3389/fpls.2026.1788062)
Supplement: Supplementary file 2 [file DataSheet2.pdf]

Integrating combining ability and multivariate analysis for the selection of superior tomato hybrids

Supplementary Table 1: Resistance profile of Italian tomato inbred lines based on SNP markers associated with major disease resistance genes. Inbred lines are grouped according to their use as Male or Female parents in the hybridization scheme.

**Diseases**	**Male**				**Female**				
	P144	P146	P241	P121	P174	P390	P115	P255	P230
Meloidogyne incognita (*Mi23*) – SNP09M3	S[1]	S	S	S	R	R	R	S	S
Fusarium oxysporum (*I-7*) – SNP93c	S	S	S	S	S	S	S	S	S
Fusarium oxysporum (*I-3*) – SNP10B	R	R	R	R	R	S	R	S	R
Fusarium oxysporum (I-*2*) – SNP13Q	R	R	R	R	R	R	R	R	R
Verticillium albo-atrum (*Ve*) – SNP16	R	R	R	R	S	S	S	R	S
Tomato spotted wilt virus (TSWV, *Sw*-7) – SNP150	R	S	S	S	R	R	S	S	R
Tomato spotted wilt virus (TSWV, *Sw-5*) – SNP25H	R	R	R	R	S	R	R	R	S
Fusarium crown & Root Rot (FCCR/Frl) – SNP38D	R	R	R	R	S	S	S	S	S
Tomato yellow leaf curl virus (TYLCV, *Ty-1*) – SNP02	S	R	R	S	S	S	S	S	S
Tomato yellow leaf curl virus (TYLCV, *Ty*-2) – SNP39B	U	S	S	S	U	S	U	R	S
Tomato yellow leaf curl virus (TYLCV, *Ty*-2) – SNP6702	S	H	H	H	S	H	H	S	H
Tomato mosaic virus (ToMV, *Tm1*) – SPQ166a	S	S	S	S	S	S	S	S	S
Tomato mosaic virus (ToMV, *Tm2*) – SNP176F	R	R	R	R	S	S	S	S	S
Tomato mosaic virus (ToMV, *Tm2a*) – SNP14G	R	R	R	R	S	S	S	S	S

[1]Susceptible (S); Resistant (R); Heterogeneous (H); and Unknown (U)

Supplementary Table 2: Estimates of general combining ability (GCA) of the parental lines and specific combining ability (SCA) of the hybrids for the evaluated traits. LL, leaf length; LW, leaf width; ID, internode diameter; IL, internode length; FS, fruit shape; PF, pericarp firmness; SSC/TA, soluble solids to titratable acidity ratio; NFP, number of fruits per plant; YPP, yield per plant; EYLD, estimated yield.

Group	Genotype/Hybrid	LL	LW	ID	IL	FS	PF	SSC/TA	NFP	YPP	EYLD
Male (GCA)	P144	-2.3115	-3.4890	0.2155	-2.6350	0.0095	-1.9690	-0.4870	11.2025	3.3860	-2.8645
	P156	-0.2755	0.5630	-0.2825	0.3010	0.0295	-1.1270	0.6030	-5.2875	-1.6660	-5.2665
	P241	2.4005	3.9610	-0.0845	0.3670	-0.0105	0.9930	0.7450	-6.6215	-1.9580	1.3735
	P121	0.1865	-1.0350	0.1515	1.9670	-0.0285	2.1030	-0.8610	0.7065	0.2380	6.7575
Female (GCA)	P174	-0.0390	2.6185	-0.5765	-0.2180	0.1785	0.4860	-0.0505	2.6020	0.8055	-0.0050
	P390	-1.2315	0.7010	0.1760	2.0320	-0.0190	-1.7940	-0.2505	7.6820	2.1980	-0.1275
	P115	-0.5715	-3.2890	0.2160	0.2020	-0.0865	-0.1090	0.6620	1.5445	0.4930	-2.6400
	P255	4.7135	3.1435	0.7185	-0.9655	-0.0440	1.5135	-1.4355	-6.5705	-1.9445	6.5650
	P230	-2.8715	-3.1740	-0.5340	-1.0505	-0.0290	-0.0965	1.0745	-5.2580	-1.5520	-3.7925
Male × Female (SCA)	H1	-4.5610	-7.9685	-0.4255	-1.4500	-0.0045	1.7740	0.2845	4.2400	1.2465	4.6370
	H2	1.1315	-3.8010	0.1620	-1.7000	-0.0070	-0.0160	0.5345	-3.8400	-1.0460	-0.4505
	H3	2.9715	0.4390	0.4520	0.8000	-0.0595	0.1590	0.5520	-7.5925	-2.3010	2.1420
	H4	1.6865	7.0065	0.2795	-0.0325	-0.0320	-0.6435	-0.0105	6.7525	1.9965	4.3070
	H5	-1.2285	4.3240	-0.4680	2.3825	0.1030	-1.2735	-1.3605	0.4400	0.1040	-10.6355
	H6	5.2330	5.8095	-0.4275	-4.0460	0.0955	-1.7980	-1.3855	-9.3800	-2.7315	-13.7310
	H7	-4.0745	-0.8530	0.6600	5.0340	-0.0070	1.1220	-1.3455	-1.2400	-0.6940	5.1115
	H8	-3.2045	-6.1130	-0.7200	-4.1360	0.0405	-1.4230	-0.9080	-6.1025	-1.7490	-9.8060
	H9	0.1505	1.8845	0.1175	1.7015	0.0080	1.5145	-0.3305	12.5725	3.8485	17.3190
	H10	1.8955	-0.7280	0.3700	1.4465	-0.1370	0.5845	3.9695	4.1500	1.3260	1.1065
	H11	-1.2730	0.5815	0.8745	3.5480	-0.1245	-0.5380	-0.0275	11.4040	3.3905	-1.4210
	H12	1.4795	-0.0010	-0.7080	-1.7020	0.0630	0.6920	0.8325	-0.9060	-0.1620	5.9915
	H13	-1.7405	2.9890	0.0820	3.4680	0.0205	0.4570	0.7200	4.5615	1.3430	5.9140
	H14	3.9745	-2.4435	-0.0805	-1.0345	0.0180	-1.1455	-0.1725	-16.8735	-5.0895	-12.2710
	H15	-2.4405	-1.1260	-0.1680	-4.2795	0.0230	0.5345	-1.3525	1.8140	0.5180	1.7865
	H16	0.6010	1.5775	-0.0215	1.9480	0.0335	0.5620	1.1285	-6.2640	-1.9055	10.5150
	H17	1.4635	4.6550	-0.1140	-1.6320	-0.0490	-1.7980	-0.0215	5.9860	1.9020	-10.6525
	H18	1.9735	2.6850	0.1860	-0.1320	-0.0015	0.8070	-0.3640	9.1335	2.7070	1.7500
	H19	-5.8115	-6.4475	-0.3165	-0.6345	0.0060	0.2745	0.5135	-2.4515	-0.7555	-9.3550
	H20	1.7735	-2.4700	0.2660	0.4505	0.0110	0.1545	-1.2565	-6.4040	-1.9480	7.7425
